# Frequency and Clinical Features of Dengue Infection in a Schoolchildren Cohort from Medellin, Colombia

**DOI:** 10.1155/2012/120496

**Published:** 2012-12-13

**Authors:** Berta Nelly Restrepo, Leidy Diana Piedrahita, Ivony Yireth Agudelo, Gabriel Parra-Henao, Jorge E. Osorio

**Affiliations:** ^1^Instituto Colombiano de Medicina Tropical, Universidad CES, Carrera 43, No. 52S99, Sabaneta, Colombia; ^2^Department of Pathobiological Sciences, School of Veterinary Medicine, University of Wisconsin-Madison, 1656 Linden Drive, Madison, WI 53706, USA

## Abstract

To determine the incidence of dengue infection, we established active surveillance of febrile episodes in a cohort of schoolchildren from three schools in Medellin, Colombia. We followed a cohort of 2,379 schoolchildren in 2010 and followed 1,840 of these children the following year. During the follow-up time, 264 schoolchildren displayed 297 febrile episodes; of these, 23 episodes (7.7%) were caused by acute dengue infection. All four dengue serotypes were found, and all of the cases were mild. The most common symptoms in the dengue cases compared with those in other febrile illness were asthenia (96% versus 87%), anorexia (78% versus 57%), rhinorrhea (65.2% versus 58%), abdominal pain (56.5% versus 47.8%), arthralgia (43% versus 33%), and positive tourniquet test (13% versus 3%). This difference was not statistically significant. Pulse was elevated, and systolic arterial pressure was lower in dengue cases compared with other febrile illness (*P* < 0.05). Mosquito indexes were determined in 8 children's houses and in the schools. *Aedes aegypti* adults were found in both households and in schools, whereas *Aedes aegypti* larvae were found only in schools. These results showed an elevated dengue frequency in children, with symptoms similar to those of other febrile illness and transmission risk in households and schools.

## 1. Introduction

Dengue virus (DENV) infection is one of the most important mosquito-borne viral diseases in the world, accounting for 50 million infections and 50,000 deaths in 112 countries annually. The disease can be caused by any of the four dengue serotypes, DENV-1 to 4, and its clinical spectrum ranges from asymptomatic form to undifferentiated fever to acute fever; the most severe form can produce hemorrhage, plasma leakage, and organ failure [1]. In 2010, the incidence of dengue in Colombia was 151,774 cases, of which 5,420 were categorized as severe by current WHO definitions [2]. All four serotypes are circulating, with DENV-2 (44%) and DENV-1 (41%) being the most frequent [3]. In Medellin, the capital of Antioquia Department, 17,632 dengue cases and 112 severe dengue cases were reported in the epidemic year of 2010 [4]. The mean age of patients with dengue was 14, and the mean age of patients with severe dengue cases was 22 years [3]. The frequency and the clinical and epidemiologic features of the disease in youth population are unknown.

Different studies around the world have reported the frequency of dengue cases in school-aged children. In a two year follow-up study in Nicaragua, incidence of dengue was reported to be 12% the first year and 6% the second year [5]. In Venezuela, an accumulated incidence of 25.8% was observed [6]. In Thailand, two studies reported incidences of dengue of 5.8 and 3.4 percents, respectively [7, 8]. In Vietnam, incidences of dengue were reported to be 11.7% in one study [9] and 41.2 to 45.0% in another [10].

One of the keys to control dengue infections is the knowledge of its magnitude. In Colombia, the incidence of dengue is only known through the patients that attend health care services. We proposed to determine the incidence, clinical, epidemiological and entomological aspects of dengue infection in schoolchildren. This study is the first one in Colombia using follow-up cohort methodology.

## 2. Material and Methods

### 2.1. Study Site and Population

**Figure 1:**
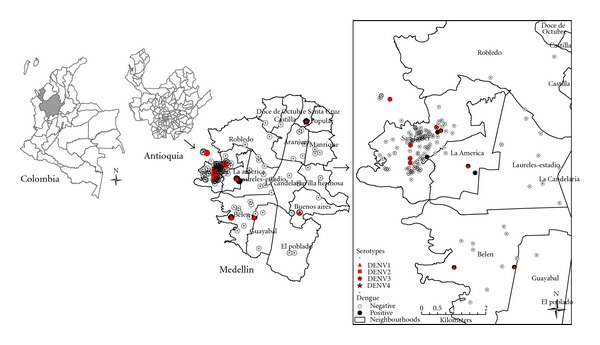
Distribution of dengue acute infection and other febrile illness in Medellin, Colombia.

This study was performed in the city of Medellin, capital of Antioquia Department, Colombia. Its population is 2,793,011 [11]. It is located 1,479 meters above sea level, and its annual average temperature is 24°C (Figure 1).

A prospective longitudinal study was conducted during May 2010 through December 2011. The study population consisted of a cohort of 2,379 students from primary and secondary schools in Medellin (three different sites located in the districts of San Javier, Poblado, and Laureles). These institutions were selected according to the following criteria: to be in the urban area, to have primary and secondary schools with a population over 2,000 students, and to have approval from school's direction.

### 2.2. Active Surveillance Cases

During the study period, dengue cases were identified by school absenteeism due to a febrile episode of less than 7 days of duration, measured by thermometer (>38°C) or reported by the parents or by the student's supervisor. A study nurse visited each school every 4 days, verifying the absenteeism of participating students. In the case of absence, students' parents were contacted to confirm the case. If the schoolchild met the inclusion criteria, a study physician performed a complete medical examination. The clinical information was recovered using a standardized case report form through a handheld computer. A serum sample (3–5 mL) was collected in the acute phase well as another serum sample (same volume) in the convalescent phase (14–21 days after the date of onset of symptoms). All serum samples were stored at−80°C until being processed. Sociodemographic data were also collected.

### 2.3. Laboratory Assays

The diagnosis testing was performed in the virology laboratory of “Instituto Colombiano de Medicina Tropical” for all symptomatic students. Dengue infection was diagnosed through of the following methods. *Serological Diagnoses*. The specific IgM antibodies were detected by DEN IgM capture ELISA (Panbio, Sinnamon Park, Australia) in serum samples taken in the acute and convalescent phases, according to the manufacturers' protocols. 

#### 2.3.1. Detection of Dengue Virus RNA by a Reverse-Transcriptase Polymerase Chain Reaction (RT-PCR)

Detection of dengue virus RNA and serotype identification was performed in all the samples collected during the acute phase according the Lanciotti et al. procedure [12] adapted by Harris et al. [13] with some modifications. Briefly, 140 *μ*L of every serum sample was used to RNA extraction using QIAamp Viral RNA kit (Qiagen, Hinden, Germany), according to the manufacturer's suggested protocol. RNA was eluted in 60 *μ*L of elution buffer and stored in −80°C until use. The extracted samples were then subjected to reverse transcription and PCR amplification. Reaction mixture contained 10 *μ*L of RNA template; the five oligonucleotide primers: D1 and TS1 at 0.5 *μ*M each, primers TS2, TS3, and DEN4 at a concentration of 0.25 *μ*M; 0.5 U of AMV RT (Promega Corp., Madison, WI, USA); 0.625 U of GoTaq Hot Start DNA polymerase; 3.5 *μ*L of 5X Green GoTaq flexi buffer; 2.6 mM of MgCL_2_ (Promega Corp., Madison, WI, USA); each of the four deoxynucleotide triphosphates at concentration of 150 *μ*M in a 35 *μ*L of reaction volume. Reverse transcription was conducted at 42°C for 60 min, followed by one cycle of initial denaturation of the reverse transcriptase and activation of the Hot Start Taq polymerase at 95°C for 5 min; 40 cycles at 95°C for 15 s, 55°C for 30 s; 72°C for 1 min and 5 min of 72°C extension. Amplification was conducted in a Bio-Rad C1000 Thermal cycler. After amplification, 10 *μ*L portion of each product was analyzed by agarose gel electrophoresis using 2% agarose in 1x TBE and a 50 bp DNA ladder (GeneRuler 50 bp DNA ladder, Fermentas, Vilnius, Lithuania). The serotype of the positive samples was determined by the amplicon size (dengue virus-1, 482 bp; dengue virus-2, 119 bp; dengue virus-3, 290 bp and dengue virus-4, 389 bp). *Isolation Virus*. From the cohort of students with a febrile episode, 17 DENV-positive samples diagnosed by any method (RT-PCR or seroconversion IgM) were randomly selected to perform dengue isolation virus using C6/36 (*Aedes albopictus*) cells with detection by direct fluorescent microscopy using anti-Flavivirus group antigen antibody [14]. The serotype of the positive samples was determined by RT-PCR. In addition, IgG antibodies to dengue virus were determined in the sample of the acute phase using DEN IgG capture ELISA (Panbio, Sinnamon Park, Australia) according to the manufacturers' protocols.

### 2.4. Definitions

Acute dengue infection was defined as a clinically suspected dengue episode with virological or serological confirmation. Serological confirmation consisted of detection of dengue IgM antibodies in acute and/or convalescent blood sample. Virological confirmation consisted of a positive test by either RT-PCR and/or virus isolation. *Secondary and primary dengue infections* were dichotomized by the detection of dengue IgG antibodies in an acute blood sample. Patients suspected of dengue infection but whose serum tests were negative for DENV were denominated as *other febrile illness (OFI)*. *Clinical classification* was made based on WHO criteria [15].

### 2.5. Mosquito Indexes

 They were determined in 8 schoolchildren's houses and in the three schools. The capture and classification methods were previously described [1, 16, 17]. Spatial distribution of the schoolchildren with febrile syndromes was mapped using the software ArcGIS 10.1 (Ersi, Redlands, CA, USA).

### 2.6. Statistical Analysis

Statistical Package for the Social Sciences, SPSS software (SPSS, version 15, Inc.01, Chicago, IL, USA) was used for data processing. Chi-squared distribution test was used for comparisons of qualitative variables between groups. Student's *t*-test was used for comparisons of normally distributed quantitative variables. Mann-Whitney test was used for comparisons of variables that were not normally distributed. Kolmogorov-Smirnov test was used as normality test. Values of *P* < 0.05 were considered significant.

The study protocol was approved by the Ethical Committee of Instituto Colombiano de Medicina Tropical and complies with Helsinki's Declaration and statement 8430 of Colombian Ministry of Health. Enrollment of students was based on a voluntary participation. Written informed consent was requested from all parents or person in charge of each school children, and written informed assent was obtained from all schoolchildren over 8 years old.

## 3. Results

Students from two public (A and B) and one private (C) schools were enrolled during the study period, leading to a prospective cohort of 2,379 children. Students ranged in age from 5 to 19 years old; the male to-female ratio was 0.9 : 1. In the second year (follow-up), 508 students withdrew and 31 new students were enrolled, for a total of 1,840 participants at the end of the study, ranging in age from 6 to 19 years old with an unchanged male to-female ratio (see Table 1).

### 3.1. Incidence and Virological Features of Dengue Acute Infections

**Figure 2:**
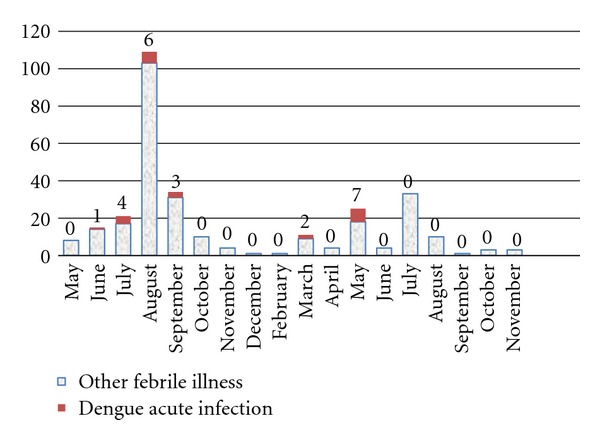
Number of dengue acute infection and other febrile illness in the cohort of schoolchildren, from May 2010 to December 2011. Medellin, Colombia.

During the study period, 297 febrile episodes illnesses were detected in 264 schoolchildren. Among them, 23 had laboratory-confirmed acute dengue infection, giving a dengue incidence in schoolchildren of 8.7% (95% CI = 5.60–12.78) and 7.7% (95% CI = 4.97–11.39) dengue incidence in febrile episode illness. In the first year, dengue incidence in febrile episode illness was 6.9% (14/202), whereas it was 9.5% (9/95) in the second year. Average days between onset of symptoms and day of examination by the physician study were 3.8 ± 1.9 days (range = 1 to 7 days). Dengue acute infection cases were reported between June and September in the first year and between March and May in the second year (see Figure 2).

All dengue cases were classified as dengue fever (WHO, 1995), and 5 of these (21.7%) were secondary dengue infections. Serological confirmation was obtained in 48% (11) of the cases, whereas 52% (12) of the cases had virological confirmation. Of the 23 cases, eleven were confirmed only with IgM antibodies, seven only with RT-PCR, two with RT-PCR and isolation virus, and three patients were confirmed by IgM antibodies, RT-PCR, and isolation virus. All four dengue serotypes were detected by RT-PCR and/or virus isolation: DENV-1 (5/23, 41.7%), DENV-2 (4/23, 33.3%), DENV-3 (2/23, 16.7%), and DENV-4 (1/23, 8.3%) (See Figure 1). In the first year (2010) of the study, only DENV-1 and DENV-3 were detected, whereas all dengue serotypes were detected in the second year (2011). The mean ± SD of days between the onset of symptoms and the drawn sample was higher in cases of dengue confirmed by IgM antibody detection than in dengue cases confirmed by either RT-PCR or isolation virus, although these differences were not statistically significant (4.1 ± 1.9 days for IgM antibodies versus 3.8 ± 1.7 days for RT-PCR and virus isolation, *P* = 0.691).

Acute dengue infections were observed in two out of three study schools. The private school (C) had the higher incidence rate (11.9%). The highest incidence rate was observed in males (compared to females) and in mestizos (in comparison with Afro-descendant ethnic group). These differences were not statistically significant (*P* = 0.589 and *P* = 0.855, resp.). We observed a higher incidence among young subjects from 5 to 9 years old (10.8%) with a lower, but not statistically significant, incidence in the older groups, 7.9% (*P* = 0.450). The age with highest incidence of dengue was the 8-year-age, 27.3% followed by the 13-year-age at 23.5% (see Table 2).

Dengue acute infections were observed in populations from all socioeconomic status, with a higher incidence rate in middle status (11.4%), *P* = 0.364 (see Table 2), and distributed across 11 neighborhoods.

### 3.2. Clinical Signs and Symptoms in Dengue Acute Infections and Other Febrile Illness (OFI) Patients

The mean age of dengue cases was 10.6 ± 3.1 (range = 6–15 years old). In comparison, the mean age of cases of other febrile illness was 11.4 ± 2.8 (range = 5–17 years old), which is not significantly different (*P* = 0.244). The clinical features of subjects with dengue infection did not present specific signs compared to subjects with OFI. Common hemorrhagic features seemed to occur more frequently in dengue acute infections than in OFI. Although there was not statistically significant difference for metrorrhagia (*P* = 0.530), there was a trend for the greater frequency in positive tourniquet tests in dengue acute infections versus OFI (*P* = 0.059). The heart rate was increased, and systolic arterial pressure was lower in acute dengue infections compared to OFI (*P* < 0.05). The clinical features of all febrile cases are shown in Table 3. In all dengue cases, most of the signs and symptoms endured until the convalescent phase, with increasing arthralgia (65% versus 43.5% in the acute phase), myalgia (65% versus 39.1% in the acute phase), back pain (40% versus 30.4% in the acute phase), vomiting (30% versus 17.4% in the acute phase), rash (30% versus 8.7% in the acute phase), bleeding gums (15% versus 4.3% in the acute phase), and epistaxis (20% versus 4.3% in the acute phase). In contrast, in OFI cases, only rash (13.9% versus 8.1% in the acute phase) and bleeding gums (13.9% versus 8.1% in the acute phase) were symptoms that increased in the convalescent phase.

None of the confirmed dengue cases were diagnosed by the study physician. The more common diagnosis for these cases was viral syndrome (69.6%), upper respiratory tract infection (17.4%), “other diagnosis” (8.7%), and diarrhea (4.3%). In the OFI group, the most common diagnosis was viral syndrome (59.3%), upper respiratory tract infection (23.7%), “other diagnosis” (6.7%), dengue (4.8%), diarrhea (4.1%), bronchitis (1.1%), and urinary tract infection (0.4%).

Only one acute dengue infection case was hospitalized. This patient presented dizziness, vomiting, generalized rash, a 14.5% increase in hematocrit, and leukopenia. The case is resolved without complications.

### 3.3. Mosquito Index and Spatial Distribution of Dengue Acute Infection Cases and OFI

The entomological indexes were determined by surveying eight households and the schools' facilities. These houses and the schools were located in the San Javier, El Poblado, and Laureles-Estadio districts of Medellin: *Aedes aegypti* adults were found in 50% of the household and 100% of the schools. *Aedes aegypti* larvae were also found in 66.6% of the schools.

The schoolchildren with febrile episodes were distributed in 12 of the 16 urban districts of Medellin. Most of the schoolchildren of the public school B were in the San Javier district, zone of influence of this school. In contrast, schoolchildren with febrile episodes from public school A and private school C were distributed throughout the whole city. The dengue infection cases were distributed in 6 districts (see Figure 1). 

## 4. Discussion

Our study has demonstrated the incidence and the clinical manifestations of acute, symptomatic dengue infection in a cohort of 2,372 schoolchildren aged 5 to 19 through the active surveillance of febrile syndromes in three urban schools of Medellin, Colombia. Between May 2010 and December 2011, 297 febrile syndrome episodes were detected in 264 schoolchildren. The frequency of febrile syndrome in 2010 and 2011 was 8.5% and 5.2%, respectively, and the incidence of dengue infection was 6.9% in 2010 and 9.5% in 2011, for a cumulative incidence during the total study period of 7.7% (23/297).

Other cohort studies in schoolchildren with surveillance of febrile episodes in different countries have reported varied results. A study conducted in Thailand between 1980-81 detected 60 febrile episodes in a cohort of 1,757 students; 5.6% of these had dengue infection [8]. Another three-year study [7] in the same country reported a 5.8% incidence of dengue infection in a cohort of 2,119 schoolchildren and was responsible for 3.2%, 7.1%, and 1.1% of the absenteeism caused by febrile illnesses, respectively, during the three years (1998 to 2000). In a rural area of Thailand, 3,055 cases of school absenteeism caused by febrile illness were observed during a two-year study (2005-6). Dengue was detected by enzyme immunoassay (EIA) test in 7.2% of these cases, (3.8% of the cases in 2005 and 11.6% in 2006) [18]. In Vietnam, in a cohort of 2,190 schoolchildren followed for four years (2004 to 2007), 690 febrile episodes in 627 children were observed and approximately 41–45% of these cases were laboratory-confirmed dengue [10]. In Nicaragua, during a two-year study with two cohorts of 467 and 719 schoolchildren, respectively, 14 and 30 children were suspected of dengue infection; of these, 4 and 6 were laboratory-confirmed dengue [5]. In Venezuela, dengue accumulative incidence in schoolchildren during two years of survey was 25.8% [6]. 

Our results are in accordance with previous results obtained in Asian countries [7–9, 18] and Nicaragua [5]. However, these results differ from data obtained in Vietnam [10] and Venezuela [6].

In our study, the incidence rate per 1,000 children from 5- to 19-year-old was 69 in 2010 and 95 in 2011. This finding was higher than the dengue incidence obtained by the official system of surveillance, 9.7 in 2010 and 0.4 in 2011 [19]. It is consistent with findings of other authors. In Nicaragua, the incidence of symptomatic dengue cases in a cohort of schoolchildren was 10 times more sensitive than the incidence reported by the surveillance system of Nicaragua's Ministry of Health [5]. In Vietnam, the incidence of dengue infection obtained through the active surveillance of febrile illness was six times more elevated than the incidence reported by the official system of surveillance [10]. Many authors highlight that the passive surveillance system usually leads to underestimation of the number of cases, leading to the misreading of the true incidence of dengue infection [5, 6, 10, 20, 21].

In our study the incidence of dengue infection was higher in men than in women, but this difference was not statistically significant. Similar findings were reported by other authors [10]. We also found a higher incidence of dengue infection in younger children (not statistically significant). While other authors did not find significant differences between age groups, they observed higher incidence rates of acute dengue disease in older children [10], and it has been hypothesized that younger age may be an additional risk factor for dengue hemorrhagic fever [8]. We observed dengue cases in all socio-economic status with predominance in the middle class, (although in this study most students were from low social-economic status). According to our findings there was no correlation between socialeconomic status and the frequency of dengue cases [22–24].

The variability in the severity of dengue disease is determined by viral factors such as serotype (e.g., DENV-1 and DENV-2 have been associated with severe dengue [10, 25, 26]), sequential infection with other serotypes [27, 28], and host genetic background [29–32]. Other studies have described that cocirculation of the four serotypes of dengue virus is related with severe dengue [33].

In our study, the circulation of all four serotypes was observed (DENV-1, 41.7%; DENV-2, 33.3%; DENV-3, 16.7%; DENV-4, 8.3%). Several authors have described the cocirculation of several serotypes in schoolchildren. Some authors reported higher frequency of DENV-4 [7, 18], others reported higher frequency of DENV-3 [6], and others DENV-2 and DENV-1 [5, 10].

We also observed differences in the frequency of the predominant serotypes depending upon the schools. In the private school C, only acute DENV-1 infection was observed, whereas all the serotypes were circulating in the public school B, with DENV-2 and DENV-3 predominating. However, the incidence of dengue infection was higher in the private school C. 

 All the confirmed dengue cases in our study were mild. This finding differs with other studies that reported severe dengue cases related to the serotypes or to the cocirculation of several serotypes [7, 8, 18]. One possible explanation of the low frequency of severe dengue cases is that only five dengue cases had sequential infection. Another possible reason is that the previous year's incidence of dengue virus infection influences the severity in the dengue disease of the subsequent years. Years with higher incidence are followed by more clinically unapparent year [7]. The population during an epidemic year of dengue can develop herd protection by the presence of heterotypic antibody. These antibodies are cross-reactive, leading to a lower disease severity [26]. On the other hand, our results are in agreement with the epidemiological reports of dengue in Medellin, where the frequency of severe dengue is approximately 0.6% of all dengue cases, and the fatality cases occur in adult patients [19]. 

Our results showed that the symptoms of dengue infection in both acute and convalescent phases are difficult to differentiate from other febrile disease. The only marked difference was the increased heart rate and lower systolic arterial pressure in dengue acute infections compared to OFI. Some authors have reported that the frequency of petechiae was associated with dengue cases [34–36]. Other authors did not find differences in the frequency of hemorrhagic signs between dengue and OFI [35, 37–39] with exception of manifestations such as melena and hematemesis which were higher in children with dengue [39]. On the other hand, tachy-brady arrhythmia has been reported during the infection by dengue virus [40]. It has also been reported that up to 29% of cardiac rhythm abnormalities can be present in children with acute dengue infection [41], with hypotension was observed in the severe cases [39]. We observed that the cardiac frequency was significantly increased and the systolic arterial pressure was significantly lowered in dengue acute infections compared to OFI. It seems important to evaluate these clinical parameters in larger samples of children with dengue compared with OFI to draw final conclusions. 

In our study, the physician did not make an accurate clinical diagnosis in the cases that were laboratory confirmed for dengue. As observed by Endy et al. [26], one of the factors for the underreporting of dengue is the mildness of the cases displaying nonspecific clinical manifestation.

The elevated entomological indexes observed in our study suggest that the risk of transmission of dengue to schoolchildren is high both in their households and in their schools. Moreover, a wide dispersion of the cases of dengue was observed in the whole city of Medellin. These results are important for the institution of prevention programs. 

## 5. Conclusions

In conclusion, we showed a high incidence of dengue virus in the school-aged population during the study period. As already described by other teams worldwide, incidence of dengue was higher than established by the local information system. This stresses the difficulty to diagnose dengue, mainly because the clinical manifestations can be similar to other infectious diseases. This kind of study is important for defining the application of the control strategies. Development of periodic monitoring of dengue through surveillance of febrile episodes would be a good tool for the early detection of outbreaks, the enforcement of vector control measures, and the management of dengue cases. Subpopulations easily accessible and restricted to well-defined areas, such as school-aged populations, may represent convenient “sentinels” for these surveys, with results that can be extrapolated to the whole community.

## Figures and Tables

**Table 1:** Sociodemographics characteristics of the cohort of schoolchildren. Medellin, Colombia.

Characteristics	2010		2011	
	No.	%	No.	%
Number of schoolchildren enrolled	2379		1840	

Sex				
Male	1123	47.2	948	51.5
Female	1256	52.8	892	48.5

Age (years)				
5	67	2.8	0	0
6	141	5.9	56	3.0
7	135	5.7	116	6.3
8	137	5.8	92	5.0
9	215	9.0	110	6.0
10	197	8.3	184	10.0
11	312	13.1	150	8.2
12	333	14.0	257	14.0
13	221	9.3	257	14.0
14	209	8.8	186	10.1
15	182	7.7	170	9.2
16	147	6.2	162	8.8
17	67	2.8	77	4.2
18	13	0.5	19	1.0
19	3	0.1	4	0.2
Mean ± SD (range)	11.3 ± 3.1 (5–19)		12.1 ± 3.0 (6–19)	

Socioeconomic status				
Low	1337	56.2	934	50.8
Median	873	36.7	759	41.3
High	163	6.9	145	7.9
No data	6	0.3	2	0.1

**Table 2:** Sociodemographics characteristics of the cohort of schoolchildren with dengue acute infection and other febrile illness, Medellin, Colombia.

	Other febrile illness		Dengue acute infection		Total	
Characteristics	*n* = 241		*n* = 23		*n* = 264	
	No.	%	No.	%	No.	%
Schools						
Public A	23	100.0	0	0.0	23	8.7
Public B	166	91.2	16	8.8	182	68.9
Private C	52	88.1	7	11.9	59	22.3

Total	241	91.3	23	8.7	264	100.0

Sex						
Male	91	90.1	10	9.9	101	38.3
Female	150	92.0	13	8.0	163	61.7

Age (years)						
5	3	100.0	0	0.0	3	1.1
6	13	76.5	4	23.5	17	6.4
7	13	92.9	1	7.1	14	5.3
8	8	72.7	3	27.3	11	4.2
9	29	100.0	0	0.0	29	11.0
10	24	92.3	2	7.7	26	9.8
11	27	93.1	2	6.9	29	11.0
12	39	92.9	3	7.1	42	15.9
13	22	84.6	4	15.4	26	9.8
14	30	93.8	2	6.3	32	12.1
15	17	89.5	2	10.5	19	7.2
16	10	100.0	0	0.0	10	3.8
17	6	100.0	0	0.0	6	2.3
Mean ± SD (range) years old	11.4±2.8 (5–17)		10.6 ± 3.1 (6–15)		11.3 ± 2.8 (5–17)	

Ethnic group						
Mestizo	227	91.2	22	8.8	249	94.3
Afrodescendant	14	93.3	1	6.7	15	5.7

Socioeconomic status					
Low	161	92.5	13	7.5	174	65.9
Medium	70	88.6	9	11.4	79	29.9
High	10	90.9	1	9.1	11	4.2

**Table 3:** Clinical features of dengue acute infection and other fever illness in schoolchildren, Medellin, Colombia.

Signs and symptoms	Other febrile illness		Dengue acute infections		Total		*P* value
	*n* = 270		*n* = 23		*n* = 293		
	No.	%	No.	%	No.	%
General status							
Asthenia	235	87.0	22	95.7	257	87.7	0.330
Muscle aches	107	39.6	9	39.1	116	39.6	1.000
Arthralgia	89	330	10	43.5	99	33.8	0.359
Back pain	74	27.4	7	30.4	81	27.6	0.809

Head							
Headache	223	82.6	19	82.6	242	82.6	1.000
Red throat	175	64.,8	18	78.3	193	65.9	0.254
Neck Pain	160	59.3	10	43.5	170	58.0	0.186
Retroorbital Pain	81	30.0	6	26.1	87	29.7	0.815
Mucosal petechiae	51	18.9	5	21.7	56	19.1	0.782
Reddened conjunctiva	14	5.2	2	8.7	16	5.5	0.363
Flushed face	13	4.8	0	0.0	13	4.4	0.609

Gastrointestinal							
Anorexia	155	57.4	18	78.3	173	59.0	0.075
Nauseas	127	47.0	10	43.5	137	46.8	0.829
Vomiting	65	24.1	4	17.4	69	23.5	0.612
Abdominal pain	129	47.8	13	56.5	142	48.5	0.516
Diarrhea	42	15.6	3	13.0	45	15.4	1.000
Hepatomegaly	2	0.7	1	4.3	3	1.0	0.218

Respiratory							
Cough	179	66.3	14	60.9	193	65.9	0.649
Nasal Congestion	172	63.7	16	69.6	188	64.2	0.656
Running nose	157	58.1	15	65.2	172	58.7	0.660
Otalgia	61	22.6	1	4.3	62	21.2	0.058

Skin							
Rash	22	8.1	2	8.7	24	8.2	1.000

Hemorrhages							
Epistaxis	26	9.6	1	4.3	27	9.2	0.707
Metrorrhagia^a^	15	11.4	1	16.7	16	11.6	0.530
Bleeding Gums	12	4.4	1	4.3	13	4.4	1.000
Positive tourniquet test	9	3.3	3	13.0	12	4.1	0.059

^a^ *n* = 65 females in other febrile illness and *n* = 69 in dengue acute infection.
